# Direct Exposure of Dry Enzymes to Atmospheric Pressure Non-Equilibrium Plasmas: The Case of Tyrosinase

**DOI:** 10.3390/ma13092181

**Published:** 2020-05-09

**Authors:** Annamaria Lapenna, Fiorenza Fanelli, Francesco Fracassi, Vincenza Armenise, Valeria Angarano, Gerardo Palazzo, Antonia Mallardi

**Affiliations:** 1 Department of Chemistry, University of Bari ‘Aldo Moro’, via Orabona 4, 70125 Bari, Italy; annamaria.lapenna@uniba.it (A.L.); francesco.fracassi@uniba.it (F.F.); vincenza.armenise@uniba.it (V.A.); Valeria.Angarano@kuleuven.be (V.A.); gerardo.palazzo@uniba.it (G.P.); 2 Center for Colloid and Surface Science (CSGI), c/o Department of Chemistry, University of Bari ‘Aldo Moro’, via Orabona 4, 70125 Bari, Italy; 3 National Research Council, Institute of Nanotechnology (CNR-NANOTEC), c/o Department of Chemistry, University of Bari ‘Aldo Moro’, via Orabona 4, 70125 Bari, Italy; 4 National Research Council, Institute for Chemical-Physical Processes (CNR-IPCF), c/o Department of Chemistry, University of Bari ‘Aldo Moro’, via Orabona 4, 70125 Bari, Italy

**Keywords:** atmospheric pressure plasma, dielectric barrier discharge, tyrosinase, enzyme immobilization, plasma chemistry, plasma etching, polymer coating, PECVD, XPS

## Abstract

The direct interaction of atmospheric pressure non-equilibrium plasmas with tyrosinase (Tyr) was investigated under typical conditions used in surface processing. Specifically, Tyr dry deposits were exposed to dielectric barrier discharges (DBDs) fed with helium, helium/oxygen, and helium/ethylene mixtures, and effects on enzyme functionality were evaluated. First of all, results show that DBDs have a measurable impact on Tyr only when experiments were carried out using very low enzyme amounts. An appreciable decrease in Tyr activity was observed upon exposure to oxygen-containing DBD. Nevertheless, the combined use of X-ray photoelectron spectroscopy and white-light vertical scanning interferometry revealed that, in this reactive environment, Tyr deposits displayed remarkable etching resistance, reasonably conferred by plasma-induced changes in their surface chemical composition as well as by their coffee-ring structure. Ethylene-containing DBDs were used to coat tyrosinase with a hydrocarbon polymer film, in order to obtain its immobilization. In particular, it was found that Tyr activity can be fully retained by properly adjusting thin film deposition conditions. All these findings enlighten a high stability of dry enzymes in various plasma environments and open new opportunities for the use of atmospheric pressure non-equilibrium plasmas in enzyme immobilization strategies.

## 1. Introduction

Enzymes are biocatalysts ubiquitous in plants, animals, and microorganisms, being able to accelerate many biochemical and chemical reactions. Their application has been therefore proposed in industrial, analytical, and biotechnological fields [1,2,3,4,5,6,7]. However, enzyme utilization is often hampered by the lack of long-term operational stability as well as by difficulties in enzyme recovery and reuse [1,2,3,4,5,6,7]. Enzyme immobilization on solid supports offers an efficient route to overcome the above limitations, as it facilitates the recovery of the biocatalyst over multiple reaction cycles [1,2,3,4,5,6,7]. Indeed, compared to their free form, immobilized enzymes generally exhibit improved stability and robustness to support applications in various domains [4,5,6,7,8,9]. Notwithstanding this, generalizable immobilization procedures are far from being assessed, because the widely different structural and functional characteristics of enzymes do not allow identifying a single procedure and solid support suitable for all enzymes and their diverse applications [5]. Moreover, each immobilization method has its own advantages and disadvantages [5,6,7,8,9,10,11,12,13,14,15,16]. Adsorption is simple, cheap, and effective, but very often reversible; covalent bonding effectively improves durability, but is expensive and easily worsens enzyme performance; encapsulation (or confinement) suffers from problems associated with mass-transfer limitations. Interestingly, recent studies have proposed the fruitful combination of two immobilization methods such as enzyme adsorption on a suitable support and subsequent overcoating with a protective thin film [17,18,19]. With this approach, the enzyme maintains sufficient conformational freedom while entrapped under the thin layer, which prevents enzyme leaching and allows the passage of substrates and products.

Over the last decades, low pressure non-equilibrium (cold) plasma processes have been exploited in a plethora of immobilization strategies [17,19,20,21,22,23,24,25,26]. They have been successfully used, for instance, (i) to properly modify the surface chemistry and morphology of various solid supports to enhance enzyme adsorption [19,20,21], (ii) to functionalize the support surface with specific chemical groups that can act as covalent binding sites for enzyme attachment [22,23,24,25], (iii) to immobilize enzyme by overcoating or entrapment with a plasma-deposited thin film [17,19,20,26].

The research in this field has received renewed impetus in recent years due to the development of atmospheric pressure (AP) plasma technology, which represents nowadays a new avenue for surface processing of materials with reduced costs and easy-to-handle apparatuses, even under open air conditions [27,28,29,30,31,32,33,34,35]. Novel strategies have been proposed for enzyme immobilization. Relevant examples include, for instance, the injection of the aerosol of an enzyme solution in the AP cold plasma to obtain either (i) the deposition of a biocomposite coating consisting of a plasma-polymerized organic matrix in which the enzyme remains entrapped [28,29], or (ii) the enzyme plasma polymerization, which leads to the deposition of a bioactive layer with no added matrix or encapsulant material [33]. It has been also demonstrated that, due to their remote operation, atmospheric pressure plasma jets offer a unique tool towards enzyme immobilization under soft reactive conditions [33]. Indeed, they can afford the desired surface modification with easier, faster, and more cost-effective plasma processes [27,32,33,34]. Moreover, considerable efforts have been devoted to investigate the direct interaction of AP non-equilibrium plasmas with various biomolecules (e.g., proteins and nucleic acids) both in the dry state and in aqueous solutions, focusing on many different biomedical, biotechnological, and pharmaceutical applications [28,35,36,37,38,39,40,41,42,43,44,45].

We recently proposed a two-step immobilization procedure in which the enzyme glucose oxidase (GOx) from *Aspergillus niger* is first deposited on a support, and subsequently coated with a polymer layer by using atmospheric pressure plasma-enhanced chemical vapor deposition (PECVD) [35]. A systematic investigation of effects of plasma exposure on GOx functionality was carried out by evaluating the influence of the enzyme amount as well as of various process parameters (e.g., plasma excitation conditions, feed gas composition, exposure time). We demonstrated that GOx activity can be preserved by proper selection of both the enzyme exposed amount and plasma processing conditions. We also envisaged that the proposed immobilization strategy could be applied also to other enzymes. However, each enzyme is expected to be sensitive in a peculiar way to plasma exposure during the immobilization procedure, which could produce changes and distortions in enzyme structure, and in turn, induce its partial or total inactivation [35]. To assess the versatility of our approach, the interaction of AP non-equilibrium plasmas with other enzymes needs therefore to be investigated.

Here, we focus our attention on mushroom tyrosinase (Tyr), i.e., a copper-containing enzyme, which in presence of oxygen catalyzes the *o*-hydroxylation of monophenols and the oxidation of *o*-diphenols into reactive *o*-quinones, which undergo spontaneous polymerization leading to dark compounds (e.g., melanine) [46,47,48]. In particular, Tyrosinase from *Agaricus bisporus* is a heterotetramer comprising two heavy (H) and two light (L) chains, with a molecular mass of about 130 kDa [47]. Its structural characteristics are very different from that of glucose oxidase from *Aspergillus niger*, which is a highly glycosylated homodimer, containing iron, with a molecular mass of about 160 kDa [49].

Specifically, in this work, very tiny amounts of Tyr from *Agaricus bisporus* were deposited on a solid support by drop casting, and after drying, exposed to atmospheric pressure dielectric barrier discharges (DBDs). To explore different scenarios that may occur when DBDs interact with the enzyme, a range of chemical environments and processing conditions typically used in surface modification was investigated. Therefore, DBDs were fed with pure helium, helium/oxygen, and helium/ethylene mixtures, to reproduce the main plasma processes relevant to surface engineering, i.e., plasma treatment, plasma etching, and PECVD, respectively. In particular, ethylene-containing DBDs led to the deposition of a hydrocarbon polymer thin film onto dry Tyr [35,50]. After plasma exposure, the enzyme was dissolved in buffer solution and its activity was spectrophotometrically followed. X-ray photoelectron spectroscopy (XPS) and white light vertical scanning interferometry (WLVSI) were used to characterize Tyr dry deposits before and after plasma exposure. The results are discussed and compared with those previously obtained for GOx [35], to gain insights into processes that can take place when DBDs interacts with dry enzymes and to enlighten new opportunities for the use of AP non-equilibrium plasmas in enzyme surface engineering.

## 2. Materials and Methods

### 2.1. Reagents

Tyrosinase from mushroom (≥ 1000 unit/mg solid) and dopamine hydrochloride were purchased from Sigma-Aldrich (Milan, Italy). Di-sodium hydrogen phosphate and sodium di-hydrogen phosphate were bought from Fluka (Rodano, Italy). Compressed helium (99.9995%) and oxygen (99.999%) gas cylinders were purchased from Sol (Monza, Italy), while ethylene (99.95%) was bought from Air Liquide Italia Service (Ostuni, Italy).

### 2.2. DBD Reactor and Plasma Processes

Plasma processes were carried out using an home-made atmospheric pressure plasma reactor with DBD electrode configuration, as described previously in full detail [35,50]. The plasma was generated by applying a sinusoidal high voltage (HV) between two parallel plate electrodes (50 × 50 mm^2^ electrode area, 4 mm gas gap), both covered with a dielectric alumina plate [35,50]. Experiments were carried out at the fixed excitation frequency (f) of 20 kHz and applied voltages (V_a_) of 0.85 and 1.1 kV_rms_ (Table 1). The electrical characterization of the DBD was accomplished with a digital oscilloscope; the voltage applied to the electrodes was measured by means of a HV probe and the total current flowing through the circuit was determined indirectly by measuring the voltage drop across a 50 Ω resistor connected in series with the ground electrode [35,50,51]. The average specific power dissipated by the discharge (P_s_) was calculated as the integral over one cycle of the product of the applied voltage and the current, divided by the period and the electrode area [35,50].

The DBD electrode system was located into an airtight Plexiglas chamber, kept at constant pressure (10^5^ Pa). Before each experiment, the chamber was purged with 6 slm (standard liters per minute) of He for 20 min to reduce air contaminations. During plasma processes, the samples were placed in the middle of the discharge region, onto the alumina plate covering the lower electrode. DBDs fed with He, He/O_2,_ and He/C_2_H_4_ mixtures were utilized. He flow rate (Φ_He_) was kept fixed at 8 slm, O_2_ concentration ([O_2_]) in the He/O_2_ mixture was equal to 1% (hereafter referred to as He/1% O_2_ mixture), ethylene concentration ([C_2_H_4_]) in He-C_2_H_4_ mixtures was varied in the range 0.1–1%. Process duration (t), also referred to as plasma exposure time, was varied between 10 and 60 min. Table 1 summarizes plasma processing conditions investigated in this study.

### 2.3. Sample Preparation, Manipulation and Enzyme Functionality Assay

**Figure 1:**
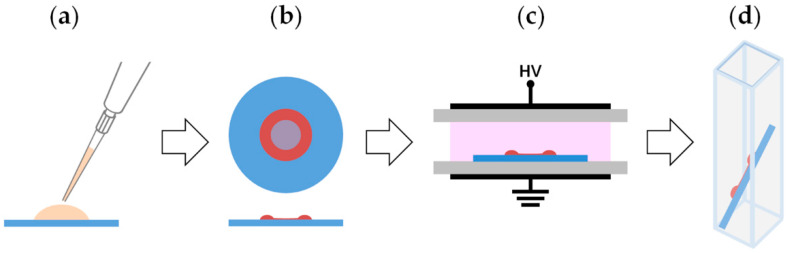
Schematic diagram summarizing the main steps of the experimental procedure used in this work: (**a**) deposition of a 5-μL drop of Tyr solution on a glass slide; (**b**) drying of the Tyr solution under reduced pressure; (**c**) exposure of the dry Tyr deposit to the atmospheric pressure dielectric barrier discharge (DBD); (**d**) Tyr solubilization in buffer solution for spectrophotometric assay.

Figure 1 summarizes the main steps of the experimental procedure utilized in this work. Five-microliter drops of enzyme solution in deionized water were deposited on round borosilicate glass slides (Figure 1a; slide diameter and thickness of 12 and 0.15 mm, respectively) and dried under reduced pressure for 30 min, leading to the formation of a ring-shaped Tyr deposit (Figure 1b). The amount of dry tyrosinase used in the experiments is expressed as micrograms of enzyme present in the 5-μL drop. A set of slides was prepared and retained to be used as control samples, while a second set was placed in the DBD reactor, to be subjected to the selected plasma process (Figure 1c). It is worth specifying that sample placement in the discharge region does not appreciably alter both gas flow dynamics and plasma behavior. This is mainly due to the fact that the support selected for this work (i.e., the glass slide) is made of a dielectric material and is both very thin (0.15 mm) and considerably thinner than the gas gap (4 mm) [52].

After that, each slide was immersed in 1 mL of buffer solution (i.e., sodium phosphate buffer) to obtain enzyme solubilization in a few seconds, under mild stirring (Figure 1d). The rapid and complete resolubilization of the enzyme in aqueous buffer indicates that interactions with glass (likely van der Walls and possibly H-bonds) are weaker than the interaction of Tyr with water. A polypropylene pipette tip was used to scratch the hydrocarbon thin film deposited on dry Tyr by helium-ethylene fed DBDs [35], allowing the release of the entrapped enzyme in the buffer solution through the lacerations made with the tip. Also, in this case the solubilization of the dried enzyme occurs in a few seconds. Control experiments showed that, after solubilization, the recovered activity of 10 μg of Tyr dried and exposed to ethylene-containing DBDs equals that recorded for the same amount of free enzyme in solution, accounting for a total enzyme recovery after scratching.

After solubilization, the enzyme activity was evaluated by a spectrophotometric assay carried out with a V-530 spectrophotometer (Jasco, Easton, PA, USA). Dopamine was used as substrate (S) and the increase in absorbance at 475 nm, due to dopaminechrome formation, was measured [48]. The assay was performed in 20 mM sodium phosphate buffer at pH 7.0, for a total volume of 1 mL. In a typical activity assay, the reaction started upon addition of 6 mM of dopamine (dopamine concentration >> Michaelis constant), then the absorbance increase was monitored. Reaction rates are expressed in absorbance min^−1^. The residual activity was determined as the percentage ratio between the reaction rate measured for the enzyme exposed to the plasma and the reaction rate measured for the same amount of enzyme dried out on a glass slide but not exposed to plasma (i.e., the control). Importantly, it was duly verified that (i) the drying procedure does not alter Tyr activity, which for the control sample is fully recovered after solubilization; (ii) no changes occur in Tyr activity when samples are placed in the DBD reactor and exposed to the He purging flow as well as to the various feed mixtures without plasma ignition. It should be also noted that the bioactivity of the dried enzyme deposit does not seem to be affected by the temperature to which it is exposed in the reactor during the plasma process (in our conditions the electrode temperature does not exceed 45 °C). Control experiments showed that, after solubilization, the recovered activity of 10 μg of Tyr dried and exposed to different plasmas for 60 min equals that recorded for the same amount of free enzyme in solution. Therefore, all changes in enzyme functionality observed in this study can be traced back to plasma exposure.

After selected plasma processes, the Michaelis–Menten plot of initial rate (V_0_) versus substrate concentration ([S]) was generated, by varying dopamine concentration between 0.1 and 6 mM. The Michaelis–Menten constant (K_m_) and the maximum velocity (V_max_) were determined. The error estimation of the parameters obtained by the fits was performed by Monte Carlo simulation using a suitable worksheet script in the OriginPro 8 SR0 v8.0724 (B724) package (OriginLab Corporation). The overall procedure is described below. The parameters obtained by fitting the set of experimental data to the Michaelis–Menten (M–M) function (Equation (1))

(1) $$V_{0}=\frac{V_{\max}\;[S]}{K_{m}+[S]},$$

were used to create “perfect” (noise free) data sets. Random noise, corresponding to the standard deviation obtained by the best-fit of the experimental data, was added to the perfect data set. The data were then fitted by a least-squares routine to Equation (1). This process was repeated 1000 times, taking care to add a different set of noise to the perfect data before each cycle. Best-fit parameters were analyzed for both the mean and standard deviation.

### 2.4. Topographical and Surface Chemical Characterization of Enzyme Deposits

White light vertical scanning interferometry (WLVSI) enabled the three-dimensional imaging of the dry enzyme deposits as well as the determination of their thickness before and after plasma exposure. Analyses were carried out using a Contour GT-K0X 3D optical microscope (Bruker, Tucson, AZ, USA) equipped with a 50x Mirau-type interferometric objective and a 0.55x camera zoom (0.23 × 0.17 mm^2^ field of view). In particular, for each sample, the thickness of the Tyr deposit was measured at four fixed locations of the ring-shaped structure (6 measurements per location). The residual thickness was determined as the percentage ratio between the mean thickness after and before plasma exposure. The thickness of the hydrocarbon polymer films deposited by He-C_2_H_4_ fed DBDs onto glass slides was determined using an Alpha-Step^®^ D-120stylus profiler (KLA-Tencor, Milpitas, CA, USA) as previously reported [35]. The deposition rate (DR) was estimated dividing the film thickness by the deposition time [50].

X-ray photoelectron spectroscopy (XPS) analyses were performed with a PHI VersaProbe II spectrometer (ULVAC-PHI, Inc., Kanagawa, Japan) using a monochromatic Al Kα X-ray source (1486.6 eV) operated at a spot size of 100 μm (power of 24.5 W). Survey (0−1400 eV) and high resolution (C 1s, O 1s, N 1s, S 2p, K 2p, Na 1s, P 2p) spectra were recorded in fixed analyzer transmission mode at pass energy of 117.4 and 46.95 eV, respectively. The spectrometer was equipped with an X-ray beam induced secondary electron imaging (SXI) system, which enabled the confident location of the analyzed spots on enzyme ring-shaped deposits. All spectra were acquired at a take-off angle of 45° with respect to the sample normal. Dual-beam charge neutralization was used during analysis to compensate charging effects. Special care was devoted to verify that no changes were induced in the samples by X-ray beam exposure. Data processing was performed using MultiPak software (Version 9.5.0.8, 30-10-2013, Ulvac-PHI, Inc., Kanagawa, Japan). The calibration of the binding energy (BE) scale was performed by fixing the position of the hydrocarbon component of the high-resolution C 1s spectrum at 285.0 ± 0.2 eV [35,50,51]. The high-resolution C 1s, O 1s, and N 1s spectra were curve fitted with mixed Gaussian-Lorentzian peaks after Shirley background subtraction, as described previously in full detail [35]. Analyses were repeated on two samples exposed to DBD in two different experiments (three analyzed points per sample). A maximum relative standard deviation of 10% was estimated on XPS surface atomic concentrations and on the peak area percentages of the curve-fitting components.

It was preliminarily verified by XPS and WLVSI that no changes occur in the surface chemical composition and morphology of the enzyme deposits after exposure to the He purging flow and to the various feed mixtures in the DBD reactor, without plasma ignition.

## 3. Results and Discussion

This study focuses on the investigation of the direct exposure of dry tyrosinase from *Agaricus bisporus* to atmospheric pressure non-equilibrium plasmas, generated in DBD configuration. The aim is to provide a clear and comprehensive picture of DBD effects on Tyr functionality over a range of chemical environments and experimental conditions typically used in surface processing of materials (Table 1). In particular, to explore different scenarios that may occur when an AP plasma interacts with the enzyme, Tyr dry deposits were exposed to DBDs fed with:

1. *Pure He*. Helium is used in large amount, as main gas, in many AP plasma processes [27,35,36,39,51,52,53,54,55,56,57]. DBDs fed by a pure noble gas (e.g., helium or argon) are often exploited for the plasma treatment of organic materials, i.e., to induce moderate changes in the surface roughness as well as in the chemical composition of the outermost layers of these materials [27,55,56,57]. Electrical excitation conditions used in this work (Table 1) fall within the operational window of a homogeneous DBD in He (i.e., DBD in glow regime) [27,52,53,54]. Figure S1a reports voltage and current signals registered under the selected conditions; as reported in the literature on homogeneous DBDs, the current signal is characterized by only one peak per half-cycle of the applied voltage and all positive (and negative) peaks exhibit almost the same shape, amplitude, and position in the cycle [54].
2. *He/1% O_2_ mixture*. Reactive oxygen species (ROSs) are effectively produced in AP non-equilibrium plasmas even at low O_2_ feed concentrations. They induce extensive surface chemical modifications as well as considerable etching of carbon-based materials with formation of volatile products [27,55]. Importantly, ROSs are widely known to cause biomolecule damage [36,38,40,43]. The investigation of this feed mixture allows also evaluating possible detrimental effects induced by the presence of a severe O_2_ contamination in the plasma. In this regard, it is worth mentioning that when plasma processes are carried out at atmospheric pressure in open-air conditions, O_2_ contamination inevitably occurs due to air entrainment into the plasma region [27,36]. The O_2_ concentration and electrical conditions used in this work (Table 1) are optimized to obtain plasma etching of polymers with uniform etching rate over the entire sample (e.g., ~55 nm·min^−1^ in case of a polyethylene-like coating deposited using a He-C_2_H_4_ fed DBDs). Under the selected conditions, the DBD operates in filamentary regime [27,52,53,54], without any surface damage due to discharge localization and filamentation. Figure S1b shows that, due to microdischarges formation, the current signal is formed by several peaks per half-cycle of the applied voltage, which are not characterized by the same amplitude and position in different cycles [54].
3. *He/C_2_H_4_ mixtures*. Atmospheric pressure plasma polymerization of ethylene leads to the deposition of a hydrocarbon polymer film (also referred to as polyethylene-like film) [50,51,58] on the dry enzyme, to enable its immobilization. The investigation of He/C_2_H_4_ fed DBDs allows, therefore, evaluating whether direct plasma interaction with Tyr during the PECVD process has a negative effect on its activity. The experimental conditions used in this work (Table 1) allow depositing a uniform and smooth polymer layer over the entire sample. As far as the coatings are concerned, by changing the ethylene concentration and the applied voltage, it is possible to vary their deposition rate from ~20 to ~40 nm·min^−1^, without significant changes in their chemical composition and morphology [35]. With regard to the discharge regime, it is worth mentioning that at the excitation frequency of 20 kHz: (i) the DBD operates in homogeneous regime when [C_2_H_4_] and V_a_ are lower or equal to 0.5% and 1.1 kV_rms_, respectively (voltage and current signals in Figure S2a,b); (ii) the DBD operates in filamentary regime when [C_2_H_4_] and V_a_ are equal to 1% and 1.1 kV_rms_, respectively. Under these latter conditions, even if the current signal is characterized by only one peak per half-cycle of the applied voltage, all positive (and negative) peaks do not have the same amplitude (Figure S2c); moreover, discharge filamentation can be clearly perceived by the naked eye.

Results obtained under experimental conditions reported in Table 1 will be described and discussed in the following subsections. First, DBDs fed with pure He and He/1% O_2_ mixture will be examined (Section 3.1); effects on Tyrosinase functionality as well as on the thickness and surface chemical composition of the dry enzyme deposits will be presented. Then, attention will shift to the impact of PECVD from ethylene-containing DBDs on Tyr activity (Section 3.2). Finally, results will be compared with data previously obtained for glucose oxidase [35], to reveal similarities and differences among the two enzymes (Section 3.3).

### 3.1. Exposure of Tyr to DBDs Fed with Pure He and He/1% O_2_ Mixture

**Figure 2:**
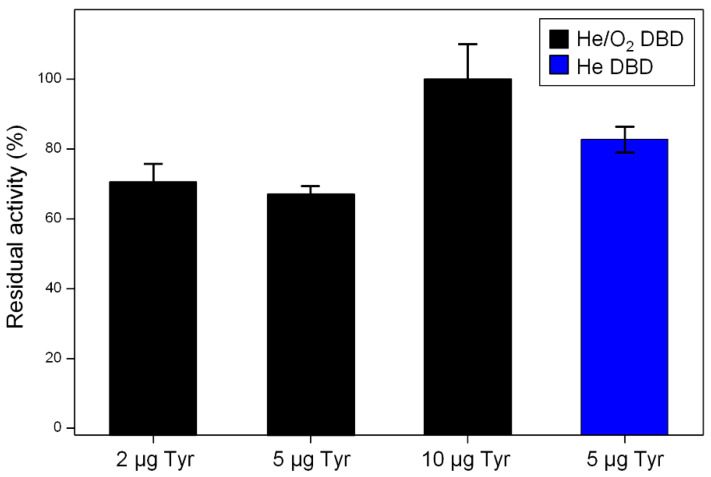
Tyrosinase residual activity after 30 min exposure to DBDs fed with He/1% O_2_ mixture (Tyr amount = 2, 5, 10 µg) and pure He (Tyr amount = 5 µg).

Plasma exposure seems to affect Tyr functionality only when very tiny amounts of enzyme are used in the experiments (i.e., 2 or 5 µg). In fact, when the amount of dry Tyr is equal to or greater than 10 µg, no activity reduction is observed even after long exposure (i.e., t > 30 min) to DBDs fed with pure He and He/1% O_2_ mixture. Results obtained for a plasma process duration of 30 min (Figure 2) can be summarized as follows: (i) the He/1% O_2_ fed DBD has no effect on Tyr activity when the exposed amount is 10 µg, while leads to similar values of residual activity in case of 2 and 5 µg of Tyr (70 ± 5% and 67 ± 2%, respectively); (ii) exposure of both 2 and 5 µg of Tyr to the pure He DBD leads to a residual activity of about 85%, greater than that observed in case of the O_2_-containing plasma, revealing that the He DBD has a lower impact on the enzyme activity than the He/1% O_2_ fed DBD.

**Figure 3:**
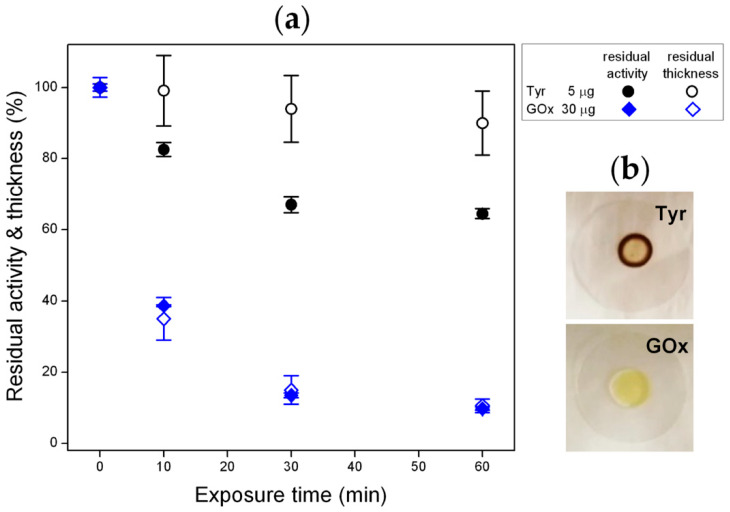
(**a**) Residual activity (closed symbols) and residual thickness (open symbols) of tyrosinase (5 µg) and glucose oxidase (30 µg) exposed to DBDs fed with He/1% O_2_ mixture for different times (f = 20 kHz, V_a_ = 1.1 kV_rms_). Glucose oxidase data are taken from Ref. [35]. (**b**) Representative pictures of Tyr and GOx deposits onto glass slides after drying (large amounts of enzyme were utilized to make the deposit morphology more visible in the pictures).

Figure 3a shows that the activity of 5 µg of tyrosinase is reduced with increasing the exposure time to the He/1% O_2_ fed DBD. In particular, Tyr residual activity decreases to ~85% upon 10 min exposure, drops to ~65% when exposure time is increased to 30 min, while remains constant for longer process duration. Very similar results are obtained as a function of the exposure time further decreasing the enzyme amount to 2 µg (Figure S3). Moreover, when the plasma treatment is carried out using a pure He DBD, a residual activity of ~85% is obtained, regardless of the process duration (Figure S3). Therefore, Tyr seems to display remarkable resistance upon plasma exposure, i.e., considerable activity retention is observed also when very low enzyme amounts are used, with very weak dependence on the exposed amount.

**Figure 4:**
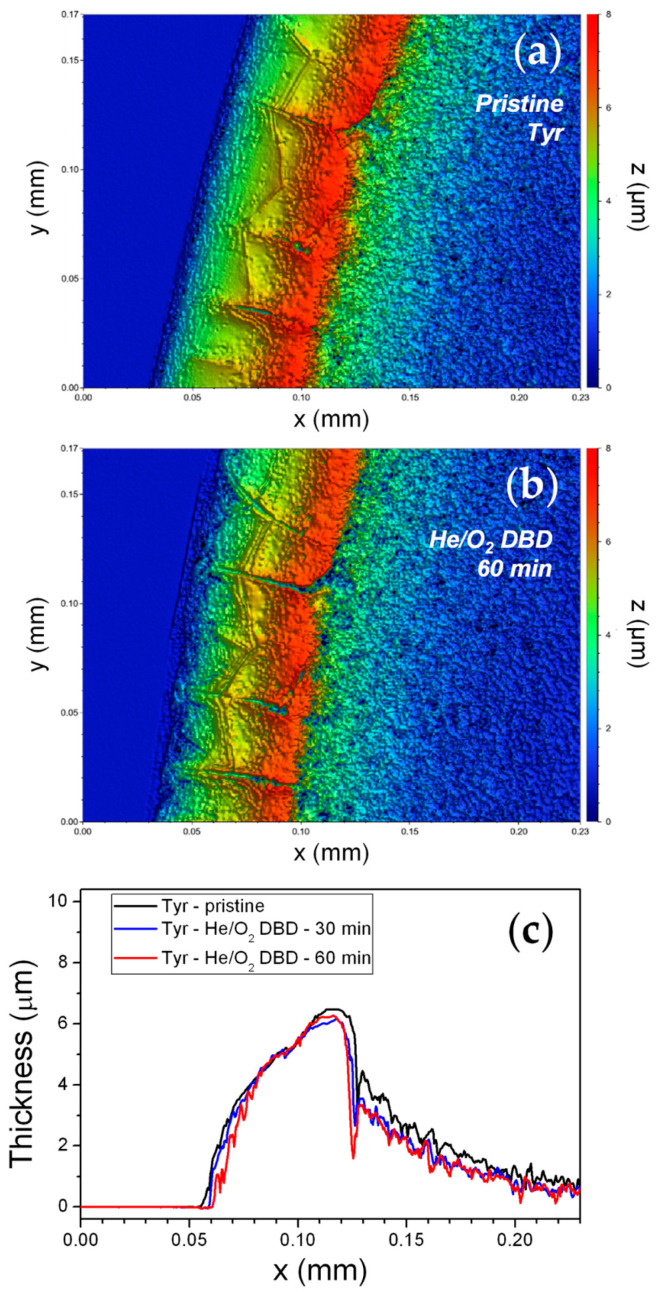
2D white light vertical scanning interferometry (WLVSI) images of a 5 µg Tyr deposit (**a**) before and (**b**) after exposure to a He/1% O_2_ fed DBD for 60 min (f = 20 kHz, V_a_ = 1.1 kV_rms_). (**c**) Cross-section thickness profiles of the Tyr deposit before and after exposure to the He/1% O_2_ fed DBD for 30 and 60 min.

Upon drying, due to the so-called coffee-ring effect, the droplet of Tyr solution deposited on the glass slide leaves a ring-shaped solid structure with a diameter of 3.4 ± 0.4 mm and a horizontal width of ~0.15 mm (Figure 3b) [40,43]. As a consequence, the major fraction of the enzyme is confined in a thick annular deposit of area 1.53 mm^2^ with an effective protein surface density (total protein amount divided by the area occupied by the coffee-ring) of 3.27 µg·mm^2^. The topography of the Tyr deposits was investigated by white light vertical scanning interferometry before and after exposure to the He/1% O_2_ fed DBD. A representative bidimensional (2D) WLVSI image of a 5-μg pristine deposit is reported in Figure 4a. Inhomogeneities and cracks are clearly visible. The height of the deposit measured on top of the coffee-ring structure is 6.5 ± 1.0 μm before plasma exposure.

The cross-section thickness profile of Figure 4c can be roughly described by a triangular shape. Taking into account that the coffee-ring thickness (t) and horizontal width (w) are much smaller than the radius (R), we can describe the deposit volume, that is given by the protein mass divided by its density (~1.3 g·mL^−1^ [59]), as the volume (V) of a triangular prism with length equal to the coffee-ring perimeter, $V=\frac{1}{2}t\cdot w\cdot 2\pi R$. The goodness of such a description can be checked by evaluating the triangular prism height from the macroscopic parameters: protein mass = 5 µg, coffee-ring radius R = 1.7 mm and width w = 0.15 mm. The calculation gives 5 µm that is within ~25% of the experimental value 6.5 µm (i.e., the maximum thickness of the deposit determined by WLVSI). Within such an approximation, the surface area exposed to the plasma (A) can be evaluated as the area of the two oblique faces, $A=4\pi R\sqrt{{\left(\frac{w}{2}\right)}^{2}+t^{2}}$, and it is possible to estimate the ratio between the exposed surface area and the overall volume (A/V) that in the case of a 5 µg Tyr deposit is 0.43 µm^−1^. The two-dimensional width and radius of the coffee-ring do not seem to vary appreciably with the deposited Tyr amount (as evaluated by the naked eye), so that the thickness of the deposit should depend mainly on the amount of protein. Within the above described approximation, 10 µg and 2 µg Tyr deposits have a maximum thickness of 8.4 µm and 1.7 µm, respectively. The corresponding A/V ratios are 0.26 µm^−1^ (for 10 µg) and 1.30 µm^−1^ (for 2 µg). Although very crude, these calculations suggest that appreciable enzyme inactivation takes place only for high enough A/V ratios. Since the A/V ratio is proportional to fraction of protein on the surface, such evidence points towards surface reactions as the main source of enzyme damage with a negligible role of others factors such as increase in temperature and exposure to UV light that take place during the plasma process, but involve the whole protein volume.

Figure 4b reports the WLVSI image acquired at the same sample position after 60 min exposure to the He/1% O_2_ fed plasma. A slight increase in the depth and width of cracks can be appreciated, along with a thickness decrease which seems to be more evident at the outer and inner rims of the deposit than at the deposit top. The filamentary character of the O_2_-contaning DBD seems not to cause localized damage of the Tyr deposit due to microdischarge formation. Cross-section thickness profiles obtained from WLVSI images (Figure 4c) show that, overall, deposit thickness weakly decreases with process duration. In particular, in case of a 5 μg Tyr deposit, a few hundred nm decrease of the average thickness is observed after 60 min, i.e., the average thickness reduction is about ~10%. Interestingly, the thickness of a polyethylene-like coating deposited in a He-C_2_H_4_ DBD decreases by 3.3 ± 0.2 µm upon exposure to the oxygen-containing DBD, under identical experimental conditions. This evidence suggests that moderate Tyr etching occurs upon O_2_-containing plasma exposure. On the other hand, the pure He DBD does not cause any morphological and thickness change in Tyr deposits.

Figure 3a reveals that, although similar trends are found for Tyr residual activity and deposit residual thickness as a function of the exposure time to the He/1% O_2_ fed DBD (5 μg Tyr), the residual thickness values are always greater than those of residual activity. For instance, after 60 min, residual thickness slightly decreases to ~90%, while residual activity drops to ~65%. It can be therefore inferred that Tyr removal via plasma etching only partially accounts for the activity reduction, and that a certain fraction of residual enzyme undergoes structural modifications upon plasma exposure, which could be responsible for inactivation and/or reduced enzyme affinity towards the substrate.

To clarify this point, the enzyme kinetic behavior was studied, determining the maximum reaction rate (V_max_) and the Michaelis–Menten constant (K_m_) values of both the control (i.e., dried enzyme not exposed to the plasma) and the dried enzyme exposed to He/1% O_2_ fed plasma for 30 min. Treatment in pure helium DBD was not considered since it scarcely affects Tyr activity.

Considering an enzyme whose initial rate V_0_ obeys the Michaelis–Menten equation (Equation (1)), where [S] denotes the initial substrate (dopamine) concentration, we have that for large [S] values, V_0_ ~ V_max_ ∝ [E] and any process that reduces the concentration of the functional enzyme [E] decreases the experimental reaction rate. On the other hand, plasma processes could also impact the affinity (of the still functional enzyme) for the substrate, leading to different values of the M–M constant.

**Figure 5:**
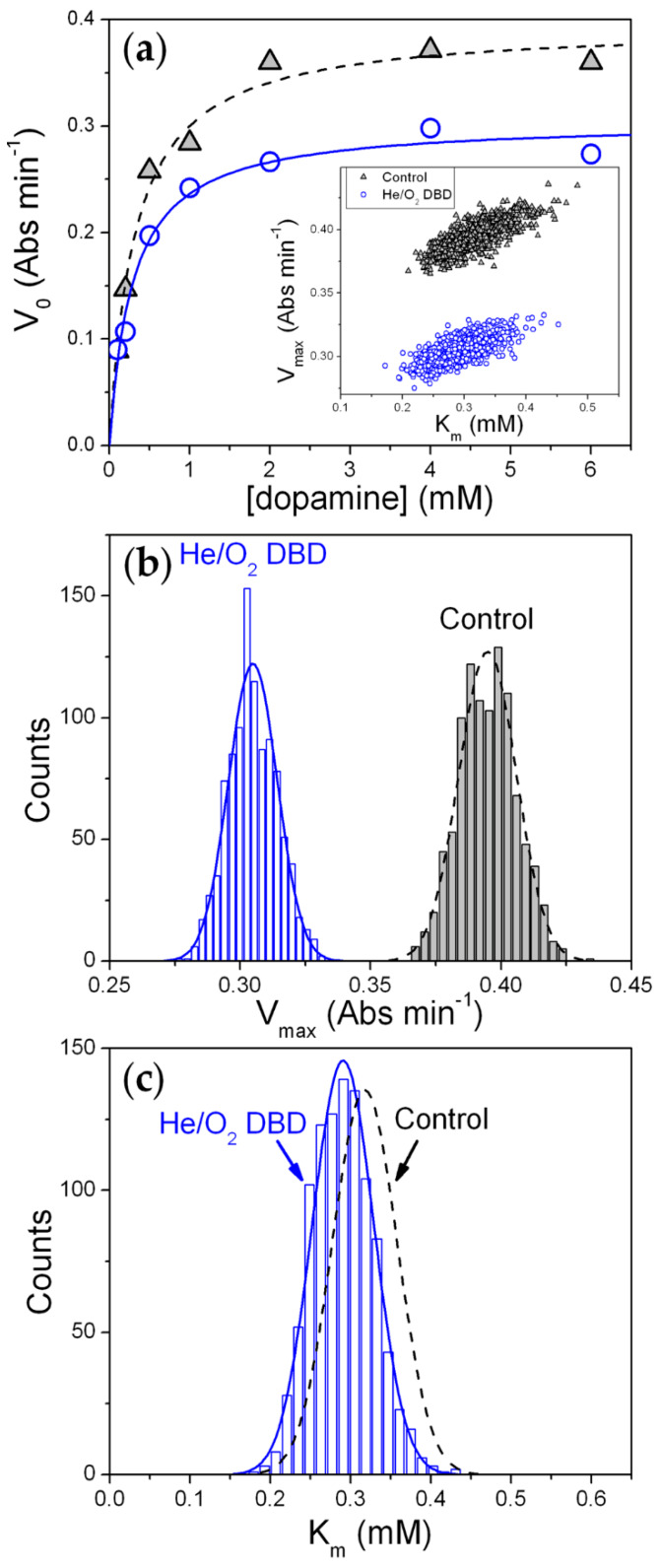
(**a**) Initial rate as a function of the dopamine concentration obtained for 5 µg of tyrosinase redissolved in buffer solution after drying (i.e., control sample; grey triangle) and after drying and exposure to He/1% O_2_ DBD for 30 min (blue circle); lines correspond to the fit to Equation (1). The results from 1000 runs of Monte Carlo analysis of best-fit data in panel (**a**) are shown in the inset of panel (**a**) (correlation between the V_max_ and K_m_ best-fit values), in panel (**b**) (frequency of the V_max_), and in panel (**c**) (frequency of the Michaelis constant, K_m_).

Representative M–M plots of the control and the plasma-treated enzyme are shown in Figure 5a. In both cases V_0_ vs. [S] plots are well accounted for by the M–M equation (Equation (1)). The best-fit values are V_max_ = 0.39 ± 0.01 Abs·min^−1^ and K_m_ = 0.32 ± 0.04 mM for the control and V_max_ = 0.30 ± 0.01 Abs·min^−1^ and K_m_ = 0.29 ± 0.04 mM for the enzyme exposed to the oxygen-containing DBD. With respect to the control, a more evident decrease (23%) in V_max_ is observed after exposure to the He/1% O_2_ fed DBD (i.e., decrease in the plateau at high [S]), while K_m_ reduction is smaller (<10%). The Monte-Carlo analysis of the best-fit of the M–M equation indicates that the 23% decrease in V_max_ is statistically relevant (Figure 5b), while the small decrease in K_m_ (Figure 5c) is likely due to the statistical positive correlation between V_max_ and K_m_ shown in the inset of Figure 5a.

Kinetic results confirm that Tyr activity reduction is greater than the thickness loss of the Tyr deposit. Moreover, the analysis of the impact of the oxygen-containing plasma on K_m_ reveals that no significant reduction of the enzyme affinity for the substrate occurs upon plasma exposure, i.e., the fraction of residual Tyr which is still functional after plasma exposure shows unchanged substrate affinity. On the other hand, there is a certain fraction of residual enzyme, which while not removed through etching, is fully inactivated by the AP plasma. Considering that, after 60 min of exposure to the He/1% O_2_ fed DBD, the residual thickness slightly decreases to ~90%, while residual activity drops to ~65% (Figure 2), the fraction of enzyme inactivated by plasma exposure can be roughly quantified as 25%.

To further investigate tyrosinase behavior, the surface chemical composition of the enzyme deposits was analyzed by XPS before and after 30 min exposure to the He/1% O_2_ fed DBD. Table 2 shows that plasma interaction with the dry Tyr deposit leads to the increase of the oxygen surface atomic concentration and consequent reduction of the carbon atomic percentage, while nitrogen surface concentration does not change significantly. Sulfur atomic percentage remains very low (1 at %); however, it is worth to note that the S 2p signal is centered at 162.5 eV for the pristine sample, as typical for C-S and S-S moieties, and shifts to 168.5 eV upon plasma exposure due to sulfur oxidation (e.g., sulfates and sulfonates formation) [60]. Interestingly, in addition to surface oxidation, considerable increase of XPS atomic concentrations of Na, K, and P is observed. This increase can be explained considering that these atomic species, present as contaminants in the pristine Tyr deposit, cannot be etched away by the oxygen-containing plasma. As reported in the literature on low pressure and atmospheric pressure plasma etching of dry proteins (e.g., bovine serum albumin [40,60]), the building up of inorganic compounds at the surface of protein deposits can provide passivation effect and limit the action of reactive oxygen species present in the plasma in terms of etching efficiency. Interestingly, the fact that the total concentration of Na, K, P, and S significantly increases from 1.5 to 11.5 at % after 30 min of plasma exposure (Table 2), and that both residual thickness and activity do not decrease appreciably when process duration exceeds 30 min (Figure 3a), suggests that the formation on the deposit surface of a layer enriched in inorganic compounds protects Tyrosinase from further etching and damage.

**Figure 6:**
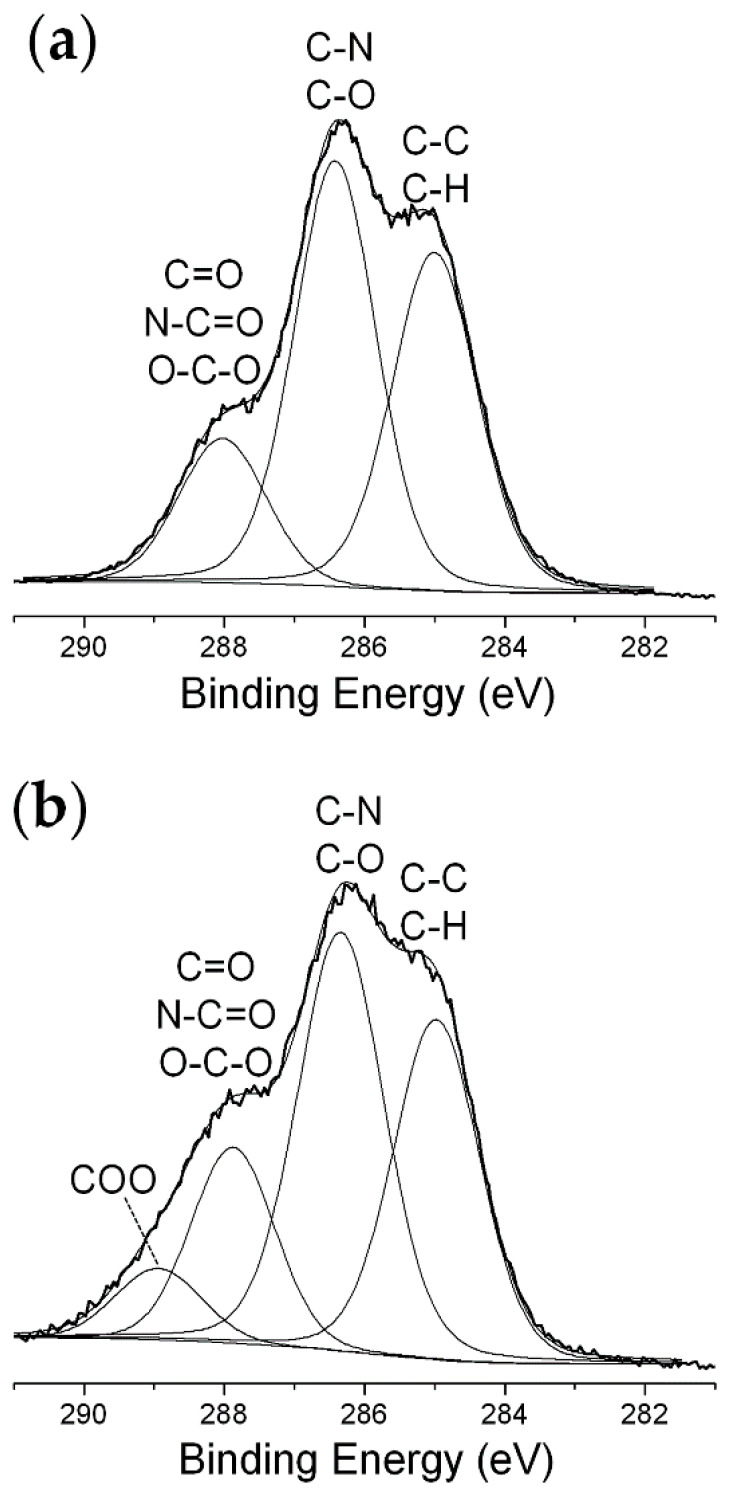
High-resolution X-ray photoelectron spectroscopy (XPS) C 1s spectra of a 5 µg Tyr deposit (**a**) before and (**b**) after exposure to a DBD fed with He/1% O_2_ mixture for 30 min (f = 20 kHz, V_a_ = 1.1 kV_rms_).

The curve fitting of the high-resolution XPS C 1s spectra (Table 3, Figure 6) shows that O_2_-containing plasma exposure induces a decrease of the peak area percentages of the hydrocarbon C-C/C-H and C-N/C-O components (as a whole, 10% decrease), a slight increase of the percentage of the component due to C=O, N-C=O, and O-C-O groups as well as the appearance of a new peak ascribed to COO functionalities (289.0 eV, 7 %) [35,40,51]. The high-resolution XPS N 1s signal of Tyr shows minor changes after plasma exposure [61], while in case of the O 1s spectrum it is possible to observe a considerable increase of the low binding energy component, ascribed for instance to O=C moieties (Figure S4, Table S1).

### 3.2. Enzyme Overcoating by a Plasma-Deposited Polyethylene-Like Film

DBDs fed with helium-ethylene mixtures allow depositing a uniform polyethylene-like coating on the dry enzyme [35]. This coating is hydrophobic (static water contact angle of ~100°) and acts as an efficient barrier, precluding the release of the enzyme upon immersion in buffer solution. When the polyethylene-like film is gently scratched, the release of the underneath enzyme is allowed and the enzyme-catalyzed reaction is immediately initiated.

The effect of PECVD from ethylene-containing DBDs on the activity of the enzyme was evaluated by varying various process parameters, such as the applied voltage (0.85 and 1.1 kV_rms_), the ethylene concentration in the feed mixture (from 0.1 to 1.0%), and the deposition time (10 and 30 min).

**Figure 7:**
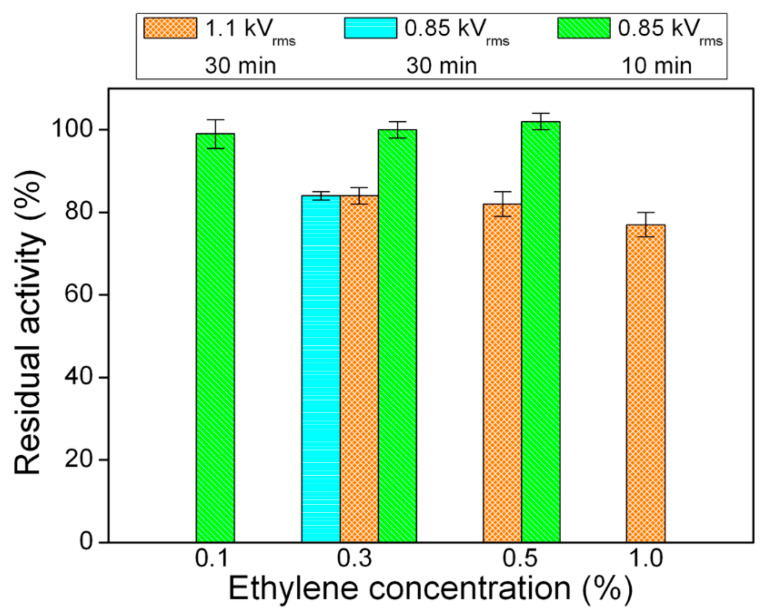
Residual activity of Tyr (5 µg) after exposure to He/C_2_H_4_ fed DBDs under different processing conditions (V_a_ = 0.85, 1.1 kV_rms_; [C_2_H_4_] = 0.1, 0.3, 0.5, 1%; t = 10, 30 min).

The exposure time turned out to be a critical parameter for preserving Tyr functionality (5 μg Tyr amount)**.** More specifically, Figure 7 shows that exposure to He/C_2_H_4_ fed DBDs for 30 min decreases Tyr residual activity to ~80%, independently from both the applied voltage and the ethylene concentration in the feed mixture. However, the reduction of the PECVD process duration to 10 min, at an applied voltage of 0.85 kV_rms_, allows fully preserving Tyr activity.

It is worth specifying that:

- The ethylene concentration in the feed mixture mainly influences the deposition rate of the coating. For instance, the DR increases from ~20 to ~30 nm·min^−1^ by increasing [C_2_H_4_] from 0.1% to 0.5%, at fixed applied voltage of 0.85 kV_rms_ (Table S2).
- The increase of the applied voltage leads to increased specific power dissipated by the plasma and slightly greater deposition rate (Table 1 and Table S2).

Considering the DR values reported in Table S2 as well as the deposition times used in the experiments (10 and 30 min, Table 1), the average thickness of the hydrocarbon coating deposited on Tyr ranges between 230 ± 10 and 1290 ± 60 nm. After thin film deposition, the surface chemical composition of the samples, as determined by XPS, is dominated by the hydrocarbon polymer. Specifically, the carbon surface atomic concentration is very high (~98%), while oxygen percentage remains very low (~2%). In addition, the high-resolution XPS C 1s signal (Figure S5) can be curve-fitted with the main aliphatic hydrocarbon component (C-C/C-H) at 285.0 eV and the very weak peak due to C-O (286.5 eV). These results can be explained considering that in all experiments the thickness of the polyethylene-like coating deposited on Tyr is considerably greater than the maximum sampling depth of XPS (~10 nm), therefore no contributions from the underneath enzyme would be expected.

The DBD regime does not seem to significantly influence residual activity results. In this regard, it is worth reminding that at the applied voltage of 1.1 kV_rms_, the discharge operates in homogeneous regime when the ethylene concentration is lower or equal to 0.5%, while a filamentary DBD is obtained for greater values of [C_2_H_4_] (i.e., 1%). Interestingly, Figure 7 shows that a very similar decrease in Tyr activity occurs after 30 min deposition at 1.1 kV_rms_, regardless the ethylene concentration and therefore the DBD regime.

### 3.3. Exposure of Dry Enzymes to DBDs: Comparison between Tyr and GOx

The aim of this section is to compare results obtained for tyrosinase, with data previously reported for glucose oxidase under identical plasma processing conditions (Table 1) [35]. This comparison allows enlightening similarities and differences between the two enzymes, obtaining a more general picture of the effect of various plasma environments and processing conditions, and finally drawing some conclusions on how experimental parameters can be optimized to achieve increased retention of enzyme activity. Table 4 compares residual activity results for GOx and Tyr after selected plasma processes.

First of all, among the investigated plasma environments, the use of a He/1% O_2_ feed mixture is found to have the highest impact on the activity of both enzymes, as described below:

- *Tyrosinase*. Considerable retention of Tyr activity is observed also when very low enzyme amounts are exposed to the plasma, with weak dependence on both the enzyme exposed amount and the process duration. The He/1% O_2_ fed DBD appreciably affects Tyr activity when the exposed amount is below 10 µg (Figure 2). For 5 µg of dry Tyr, residual activity drops to ~65% after 30 min and remains constant for longer exposure times (Figure 3a). After 60 min of exposure to the O_2_-containing DBD, the 5 µg Tyr deposit shows an average thickness loss of a few hundred nm, corresponding to a residual thickness of ~90% (Figure 3a and Figure 4c). This is consistent with a remarkable etching resistance of Tyr deposits. Both residual activity and thickness decrease with exposure time (Figure 3a); however, residual thickness values remain greater than the corresponding values of residual activity. In this regard, it is found that, after plasma exposure, the major fraction of the residual enzyme exhibits unchanged functionality and substrate affinity (i.e., the slight decrease in M–M constant is found to not be statistically relevant as shown in Figure 5c), while a minor fraction is fully inactivated. The oxygen-containing DBD leads to the formation on the deposit surface of a layer enriched in inorganic compounds. This layer seems to protect tyrosinase from etching and functional damage, limiting the reduction in Tyr activity with exposure time.
- *Glucose oxidase*. Also for GOx it is possible to identify a threshold value of exposed amount, below which the He/1% O_2_ fed DBD has an appreciable impact on enzyme activity. However, in case of GOx, this threshold value is found to be 150 μg [35], i.e., fifteen times greater than that of Tyr. Moreover, a remarkable decrease in GOx residual activity is observed when the exposed amount is reduced; for instance, after 30 min plasma exposure, residual activity steeply decreases from 85% to less than 5% by decreasing the exposed amount from 100 to 10 μg. Figure 3a represents a fair comparison between the two enzymes, because although the GOx amount used in the experiments is larger (30 μg), it spreads uniformly on a larger area (8.55 mm^2^) so that the effective protein surface density (3.51 µg·mm^−2^) is very close to the surface density of Tyr in the coffee-ring (3.27 µg·mm^−2^). It is clear from Figure 3a that GOx residual activity more significantly decreases with exposure time, and is lower than that of Tyr. On the other hand, when GOx amount is 100 μg (i.e., a thicker enzyme deposit is exposed to the plasma), residual activity slightly decreases to ~85% after 10 min and remains constant for longer exposure times. Interestingly, in case of GOx, very good agreement is found between the values of the residual activity and residual thickness (Figure 3a). Therefore, the loss in GOx activity upon exposure to the He/1% O_2_ fed DBD seems to be directly correlated to the deposit thickness decrease due to etching. Moreover, it is worth mentioning that the thickness of a 30 μg GOx deposit decreases by ~3 μm after 60 min exposure to the O_2_-containing plasma, similarly to the polyethylene-like coating. Also for GOx, the chemical modification of the deposit surface upon plasma exposure leads to the formation of a protective layer with strong ablation resistance, which progressively reduces the etching rate with increasing the exposure time [35]. Specifically, XPS results show that the O_2_-containing DBD causes a decrease of both C and N surface atomic concentrations as well as considerable increase of the atomic percentages of O, K, and Na [35]. The GOx kinetic response to the O_2_-containing plasma is different from that shown by Tyr in similar conditions. As reported in our previous paper [35], after GOx exposure to the He/1% O_2_ fed DBD: (i) the decrease in V_max_ (in agreement with the observed reduction in activity) corresponds to the thickness loss of the deposit, accounting for enzyme removal by plasma etching; (ii) an increase in K_m_ is observed, suggesting that the plasma reduces the affinity for the substrate of the still functional enzyme.

It can be, therefore, concluded that the trends observed for the two enzymes are rather similar; however, in the case of Tyr it is possible to use much smaller enzyme amounts, while maintaining greater values of both residual activity and residual thickness. GOx is much more sensitive than Tyr to the He/1% O_2_ fed DBD in terms of both protein removal through plasma etching and loss in activity and substrate affinity.

It is not trivial to explain this evidence; however, the following factors could contribute to the diverse enzyme response: (i) the different chemical structure of the two enzymes, considering that, for instance, the glycosylated structure of GOx could be more sensitive to chemical reactions occurring in the plasma, and in turn, be more prone to plasma etching; (ii) the presence of slightly different inorganic contaminations in the dry deposits, which could lead to the formation of a protective layer in different exposure times and with different composition (and likely different passivation efficacy); (iii) the morphology of the dry enzyme deposit. This last point deserves further discussion. Figure 3b clearly shows the very different structure of the Tyr and GOx dry deposits. While a ring-shaped structure is obtained for Tyr, the drying of the GOx solution drop on the glass slide leads to a uniform and roughly circular deposit with a diameter of 3.3 ± 0.4 mm (Figure 3b). Since GOx distributes over a larger area compared to Tyr, a greater fraction of the GOx exposed amount is expected to interact with the plasma. This could, at least partially, explain the more significant and comparable reduction of residual activity and thickness observed for GOx. In case of Tyr, the formation of a coffee-ring structure, leads to a reduced surface area of the deposit exposed to plasma, reasonably limiting the extent of the plasma-enzyme interaction. Even if, it is not possible to exclude that thickness loss may be underestimated in case of Tyr, due to the fact that etching mainly occurs at the outer and inner rims and in the cracks of the coffee-ring structure (Figure 4), WLVSI images clearly evidence the greater etching resistance of Tyr deposits with respect to GOx ones [35].

The above discussion suggests that enhanced retention of enzyme activity in He/O_2_ fed DBDs could be achieved in different ways: (i) by increasing the amount of exposed enzyme, and therefore, the initial thickness of the enzyme deposit (as reported in Ref. [35]); (ii) by acting on the morphology of the protein deposit and guiding, when possible, the formation of a coffee-ring structure; this latter allows reducing plasma-enzyme interaction and increasing deposit thickness, while keeping fixed the enzyme amount used in the experiments; (iii) by incorporating in the enzyme deposits very low amounts of inorganic compounds with strong resistance against plasma etching.

Results obtained in case of He/C_2_H_4_ fed DBDs show that the activity of both enzymes is not considerably affected by PECVD process conditions (Table 4). In particular, the residual activity of Tyr and GOx remains greater than ~80% and ~90%, respectively. Enzyme activity can be fully preserved by decreasing the applied voltage to 0.85 kV_rms_ and the exposure time to 10 min. These outcomes allow envisaging the possibility of using PECVD at atmospheric pressure for enzyme immobilization by overcoating, with considerable retention of enzyme functionality upon plasma exposure.

Finally, activity of both Tyr and GOx is barely affected by direct exposure to DBDs fed with pure helium (Table 4). This evidence suggests that the chemical environment generated in the plasma, due to the presence of reactive additives in the feed mixture, could play a major role in the direct interaction of DBDs with dry enzymes.

## 4. Conclusions

The direct interaction of dry tyrosinase with atmospheric pressure DBDs fed with helium, helium/oxygen, and helium/ethylene mixtures was investigated to provide a comprehensive picture of plasma effects on Tyr over a range of chemical environments and processing conditions typically used in surface modification.

First of all, results show good retention of Tyr activity also when very low enzyme amounts are exposed to the plasma (<10 μg), with a slight dependence on the exposed amount and exposure time. For 5 μg of Tyr, an appreciable decrease in enzyme activity is observed upon exposure to oxygen-containing DBD due to both enzyme etching and damage. Nevertheless, Tyr deposits display remarkable etching resistance, reasonably conferred by plasma-induced changes in their surface chemical composition and by their coffee-ring structure. In particular, XPS results show that exposure to He/1% O_2_ fed DBD leads to the formation of a surface layer enriched in inorganic compounds, which limits Tyrosinase etching and damage. Ethylene-containing DBDs allow coating Tyr with a hydrocarbon polymer film, in order to obtain its immobilization. It is found that Tyr activity can be fully preserved by properly adjusting PECVD process conditions. All these findings, along with previous data obtained for GOx, enlighten a high stability of dry enzymes in various plasma environments and emphasize the need for optimal selection of processing conditions. Future work will involve extending our investigations to a less expensive main gas (e.g., argon or nitrogen rather than helium), taking due account of the fact that plasma characteristics and optimal processing conditions inevitably change as a function of the main gas utilized.

Overall, this study suggests new opportunities for the use of atmospheric pressure non-equilibrium plasmas in enzyme immobilization strategies. For instance, in multi-step immobilization procedures AP plasma-based processes could be exploited for enzyme overcoating, interface modification, and surface cleaning, with considerable retention of enzyme activity upon plasma exposure.

## Figures and Tables

**Table 1:** Plasma processing conditions investigated in the present work.

Feed Mixture	f (kHz)	V_a_ (kV_rms_)	P_s_ (W·cm^−2^)	Φ_He_ (slm)	[O_2_] (%)	[C_2_H_4_] (%)	t (min)
He	20	1.10	0.35 ± 0.04	8	-	-	10, 30, 60
He/O_2_	20	1.10	0.40 ± 0.04	8	1.0	-	10, 30, 60
He/C_2_H_4_	20	1.10	0.40 ± 0.04	8	-	0.3, 0.5, 1.0	30
He/C_2_H_4_	20	0.85	0.25 ± 0.05	8	-	0.3	30
He-C_2_H_4_	20	0.85	0.25 ± 0.05	8	-	0.1, 0.3, 0.5	10

**Table 2:** XPS surface atomic concentrations of the dry Tyr deposit before and after 30 min exposure to the DBD fed with He/1% O_2_ mixture (f = 20 kHz, V_a_ = 1.1 kV_rms_).

	C at %	O at %	N at %	S at %	P at %	K, Na at %
Pristine Tyr - control	64	26	8.5	0.5	-	1.0
Tyr - He/1% O_2_ DBD	44	37	7.5	1.0	3.0	7.5

**Table 3:** Curve fitting results of high-resolution C 1s XPS spectra of the dry Tyr deposit before and after 30 min exposure to the DBD fed with He/1% O_2_ mixture (f = 20 kHz, V_a_ = 1.1 kV_rms_).

	C-C/C-H Peak Area %	C-N/C-O Peak Area %	C=O/N-C=O/O-C-O Peak Area %	COO Peak Area %
	285.0 ± 0.2 eV	286.3 ± 0.2 eV	288.1 ± 0.2 eV	289.0 ± 0.2 eV
Pristine Tyr - control	37	46	17	-
Tyr - He/1% O_2_ DBD	32	41	20	7

**Table 4:** Tyr and GOx residual activity after selected plasma processes (complete plasma processing conditions are reported in Table 1). GOx data are taken from Ref. [35].

			Tyr 2 μg	Tyr 5 μg	GOx 30 μg	GOx 100 μg
Feed Mixture	V_a_ (kV_rms_)	t (min)	Res. Activity (%)	Res. Activity (%)	Res. Activity (%)	Res. Activity (%)
He	1.10	30	85 ± 4	83 ± 4	95 ± 6	100 ± 6
He/1% O_2_	1.10	30	70 ± 5	67 ± 2	14.0 ± 1.0	85 ± 2
He/0.3%C_2_H_4_	1.10	30	-	84 ± 2	96 ± 3	-
He/0.3%C_2_H_4_	0.85	30	-	84.0 ± 1.0	101 ± 2	-
He/0.3%C_2_H_4_	0.85	10	-	100 ± 2	100 ± 2	-

## Supplementary Materials

The following are available online at https://www.mdpi.com/1996-1944/13/9/2181/s1, Figure S1: voltage and current signals of (**a**) a pure He DBD and (**b**) a He/1% O2 fed DBD (f = 20 kHz, Va = 1.1 kVrms), Figure S2: voltage and current signals of He/C_2_H_4_ fed DBDs generated at 20 kHz under different experimental conditions (Table 1): (**a**) [C_2_H_4_] = 0.3%, V_a_ = 0.85 kV_rms_; (**b**) [C_2_H_4_] = 0.3%, V_a_ = 1.1 kV_rms_; (**c**) [C_2_H_4_] = 1%, V_a_ = 1.1 kV_rms_, Figure S3: residual activity of tyrosinase (2 and 5 μg) exposed to DBDs fed with He and He/1% O_2_ mixture as a function of the exposure time, Figure S4: high-resolution XPS C 1s, O 1s and N 1s spectra of a 5 μg Tyr deposit before and after exposure to a DBD fed with He/1% O_2_ mixture for 30 min, Table S1: curve fitting results of high-resolution C 1s, O 1s and N 1s XPS spectra of a 5 μg Tyr deposit before and after 30 min exposure to the DBD fed with He/1% O_2_ mixture, Table S2: deposition rate (DR) of the polyethylene-like coating under the PECVD conditions investigated in the present work, Figure S5: high-resolution XPS C 1s spectrum of the polyethylene-like coating deposited on Tyr (5 µg) by using a He/0.1% C_2_H_4_ fed DBD (f = 20 kHz, V_a_ = 0.85 kV_rms_, t = 10 min, thickness of the coating = 230 ± 10 nm).
