# Supplementary material for: Direct Exposure of Dry Enzymes to Atmospheric Pressure Non-Equilibrium Plasmas: The Case of Tyrosinase
Source: Materials (Basel). 2020 May 9;13(9):2181. doi: 10.3390/ma13092181 (PMC7254212; doi:10.3390/ma13092181)
Supplement: Supplementary file 1 [file materials-13-02181-s001.pdf]

materials

Supplementary Material

Direct Exposure of Dry Enzymes to Atmospheric Pressure Non-Equilibrium Plasmas: The Case of Tyrosinase

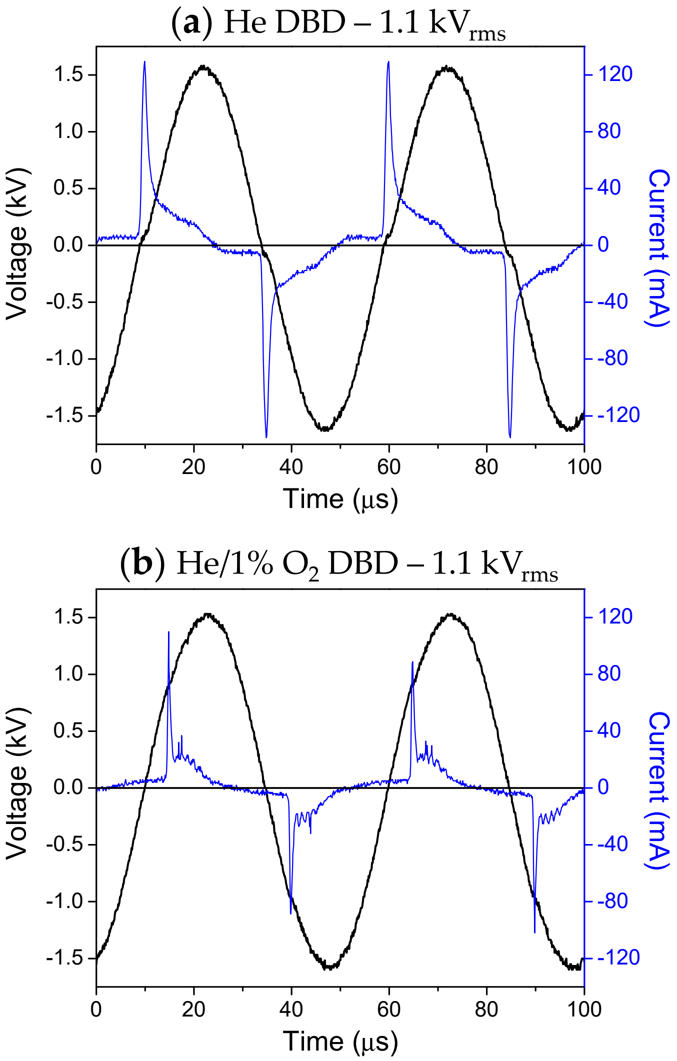

Figure S1. Voltage and current signals of (**a**) a pure He DBD and (**b**) a He/1% O_2 fed DBD (f = 20 kHz, V_a = 1.1 kV_{rms}).

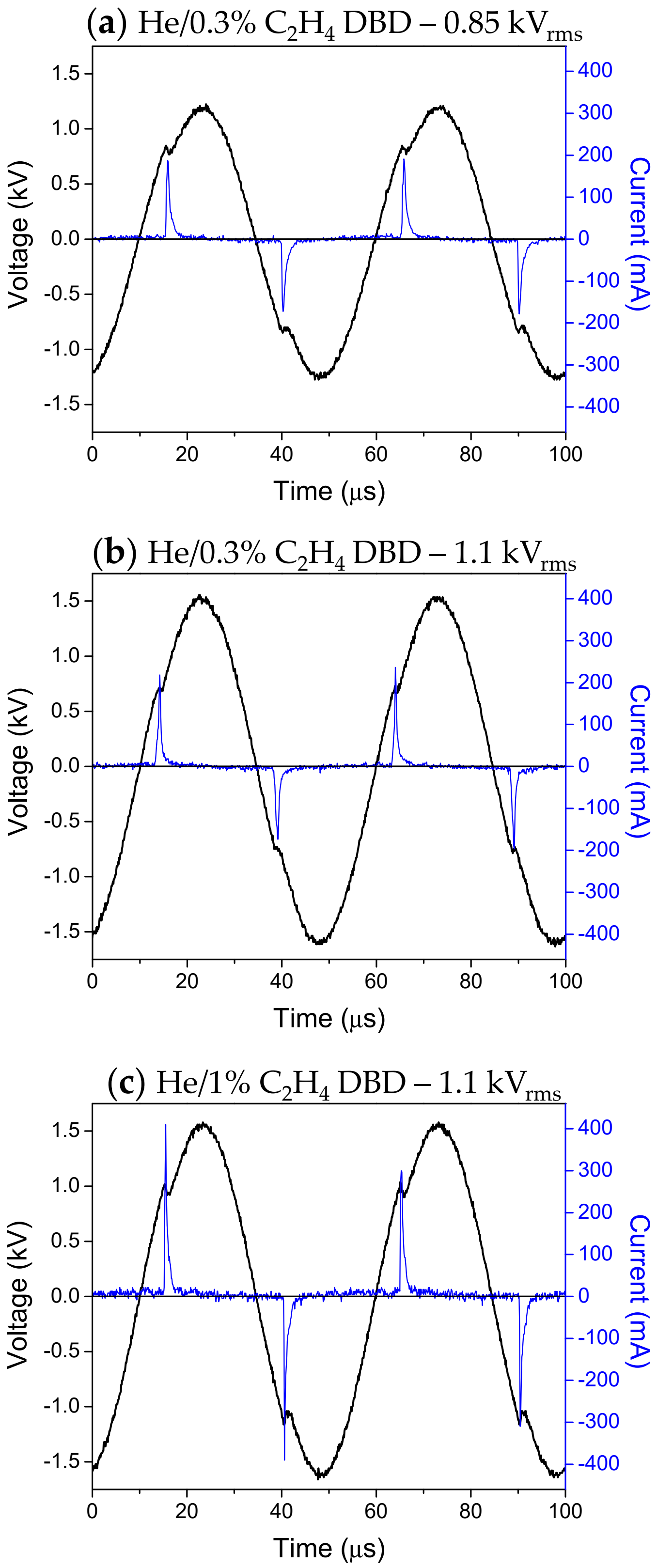

Figure S2. Voltage and current signals of He/C_2H_4 fed DBDs generated at 20 kHz under different experimental conditions (Table 1): (**a**) [C_2H_4] = 0.3%, V_a = 0.85 kV_{rms}; (**b**) [C_2H_4] = 0.3%, V_a = 1.1 kV_{rms}; (**c**) [C_2H_4] = 1%, V_a = 1.1 kV_{rms}.

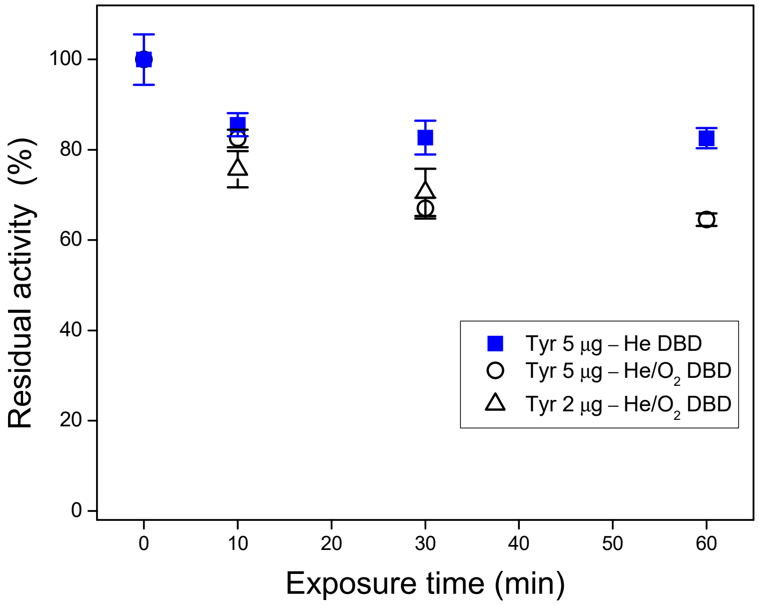

Figure S3. Residual activity of tyrosinase (2 and 5 μg) exposed to DBDs fed with He and He/1% O_2 mixture as a function of the exposure time (f = 20 kHz, V_a = 1.1 kV_{rms}).

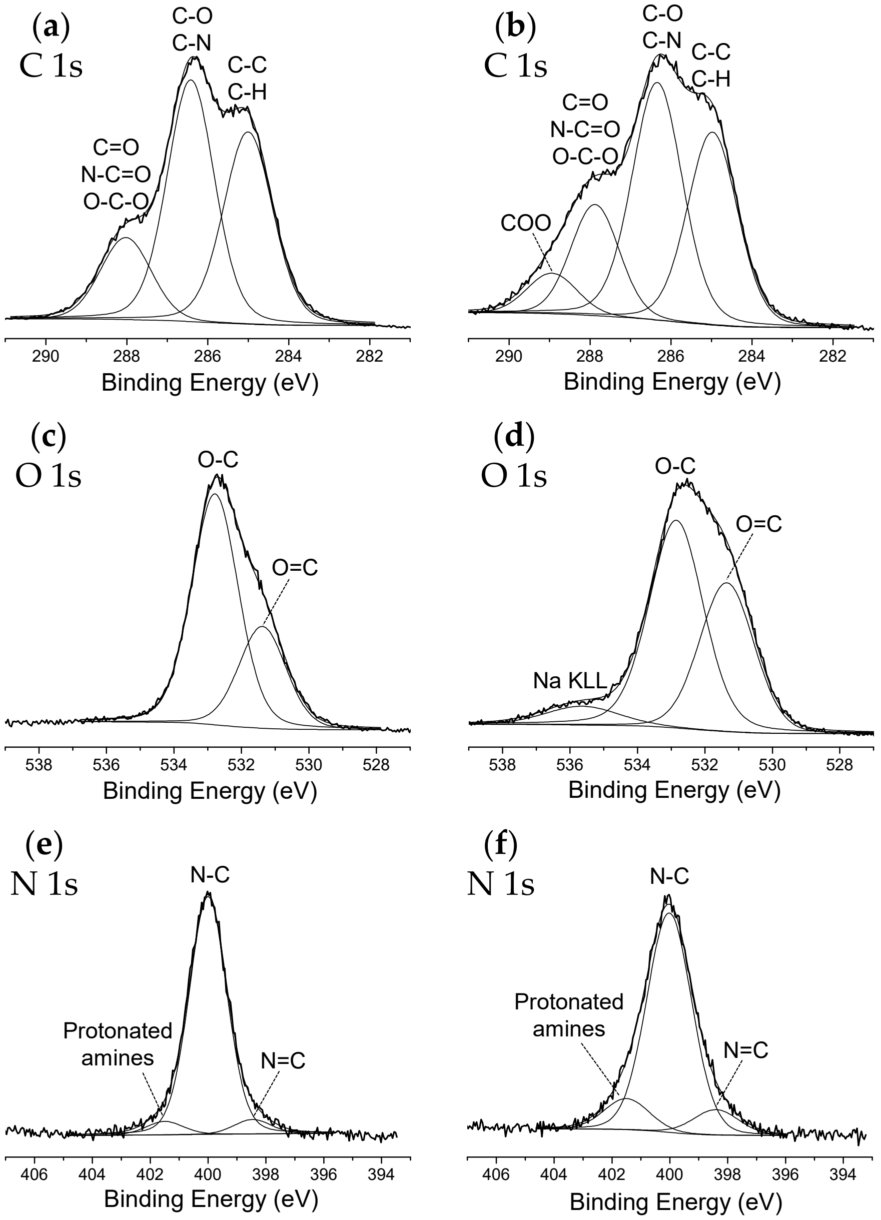

Figure S4. High-resolution XPS C 1s, O 1s and N 1s spectra of a 5 μg Tyr deposit (**a**)-(**c**)-(**e**) before and (**b**)-(**d**)-(**f**) after exposure to a DBD fed with He/1% O_2 mixture for 30 min (f = 20 kHz, V_a = 1.1 kV_{rms}).

Table S1. Curve fitting results of high-resolution C 1s, O 1s and N 1s XPS spectra of the a 5 μg Tyr deposit before and after 30 min exposure to a DBD fed with He/1% O_2 mixture (f = 20 kHz, V_a = 1.1 kV_{rms}).

		**Pristine Tyr - Control**	**Tyr - He/1% O_2 DBD**
**Component Assignment**	**Position (eV)**	**Component Peak Area %**	**Component Peak Area %**
**C-C/C-H**	284.8 ± 0.2	37	32
**C-N/C-O**	286.3 ± 0.2	46	41
**C=O/N-C=O/O-C-O**	288.1 ± 0.2	17	20
**COO**	289.0 ± 0.2	-	7
**O=C**	531.4 ± 0.2	31	45
**O-C**	532.8 ± 0.2	69	55
**N=C**	398.4 ± 0.2	5	9
**N-C**	400.0 ± 0.2	90	79
**Protonated amine groups**	401.5 ± 0.2	5	12

Table S2. Deposition rate (DR) of the polyethylene-like coating under the PECVD conditions investigated in the present work.

Feed Mixture	f (kHz)	V_a (kV_{rms})	P_s ($W \cdot cm^{-2}$)	Φ_{He} (slm)	[C_2H_4] (%)	DBD Regime	DR ($nm \cdot min^{-1}$)
He/C_2H_4	20	0.85	0.25 ± 0.05	8	0.1	Homogeneous	23.0 ± 1.0
He/C_2H_4	20	0.85	0.25 ± 0.05	8	0.3	Homogeneous	27 ± 2
He-C_2H_4	20	0.85	0.25 ± 0.05	8	0.5	Homogeneous	31 ± 2
He/C_2H_4	20	1.10	0.40 ± 0.04	8	0.3	Homogeneous	30 ± 3
He/C_2H_4	20	1.10	0.40 ± 0.04	8	0.5	Homogeneous	34 ± 3
He-C_2H_4	20	1.10	0.40 ± 0.04	8	1.0	Filamentary	43 ± 2

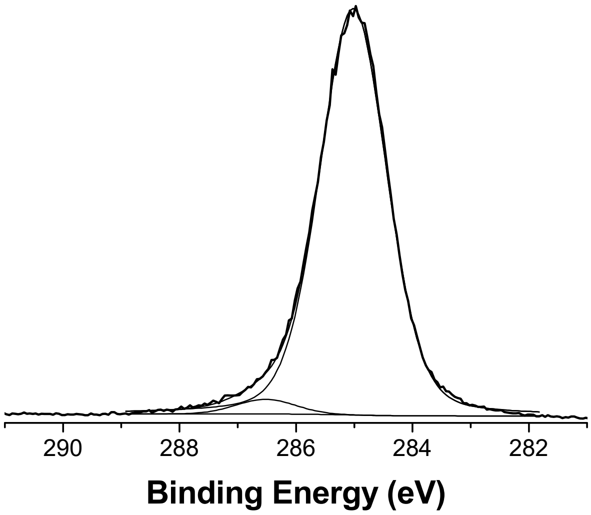

Figure S5. High-resolution XPS C 1s spectrum of the polyethylene-like coating deposited on Tyr (5 μg) by using a He/0.1% C_2H_4 fed DBD (f = 20 kHz, V_a = 0.85 kV_{rms}, t = 10 min, thickness of the coating = 230 ± 10 nm).

© 2020 by the authors. Licensee MDPI, Basel, Switzerland. This article is an open access article distributed under the terms and conditions of the Creative Commons Attribution (CC BY) license (http://creativecommons.org/licenses/by/4.0/).
